# Determining the level of resilience amongst critical care transport professionals in a fixed-wing air ambulance environment

**DOI:** 10.1016/j.afjem.2026.100966

**Published:** 2026-03-31

**Authors:** Karien Basson, Isabel Coetzee-Prinsloo, Annatjie van der Wath, Andries Masenge

**Affiliations:** a University of Pretoria, Faculty of Health Sciences, Department of Nursing Science, Pretoria, South Africa; b University of Pretoria, Department of statistics, Hatfield campus, South Africa; c Air Rescue Africa, InternationalSOS, Johannesburg, South Africa

**Keywords:** Africa, Air ambulance, Critical care transport, Resilience

## Abstract

**Introduction:**

Critical care transport within a fixed-wing air ambulance environment is characterised by its demanding and unpredictable nature, distinguishing it from conventional hospital environments. Critical care transport professionals must exhibit resilience to effectively fulfil their responsibilities and excel under stressful conditions. This study examined the level of resilience amongst flight nurses, doctors, and paramedics operating in a fixed-wing air ambulance environment in Africa.

**Methods:**

A quantitative, cross-sectional, descriptive study was conducted using a self-administered survey incorporating the Connor–Davidson Resilience Scale (CD-RISC-25).

**Results:**

A total of 48 responses were collected in 2024 from five fixed-wing air-ambulance organisations based in South Africa. Seven constructs related to resilience were measured: hardiness, coping, adaptability and flexibility, meaningfulness and purpose, optimism, emotion regulation, and cognition and self-efficacy.

**Conclusion:**

The findings suggest that participants generally demonstrated a high level of resilience. Mean scores on the CD-RISC subscales ranged from 6.52 to 22.81, with standard deviations ranging from 1.07 to 3.44. No significant difference in resilience levels was observed between teams comprising mixed professions and those of the same profession. However, optimism was more pronounced in mixed-profession teams than in same-profession teams.

**African relevance**

• Africa presents significant challenges, making it a demanding environment.
• Africa requires timely and high-quality interfacility transfers.
• Air ambulances play a crucial role in transporting patients to specialised medical facilities. These aircraft operate as airborne intensive care units, equipped with life support systems and staffed by trained critical care transport professionals who provide critical care to patients.
• Critical care transport professionals operating in the demanding environment of air ambulances, particularly in Africa, must demonstrate resilience.

Alt-text: Unlabelled box dummy alt text

## Introduction

Resilience is the capacity to endure, adapt to, and recover from challenges and setbacks [1]. It encompasses the ability to rebound from adversity while maintaining well-being and effectively functioning in difficult situations. Resilient individuals are better equipped to manage stress and employ strategies to overcome obstacles [2]. Developing skills to navigate and thrive despite difficulties is central to resilience [[2], [3], [4]].

Africa is the second largest and second most populous continent in the world. Despite its abundant natural resources, it remains the poorest continent, characterised by significant regional disparities. While countries such as South Africa, Kenya, and Tunisia possess recognised healthcare systems, medical services remain inaccessible in numerous areas. Development plans are intended to address these healthcare gaps across diverse regions. Consequently, given the high disease and mortality rates, air ambulances have become crucial for providing access to improved healthcare services [3,5].

Air ambulances facilitate both domestic and international patient transfers across Africa, thereby increasing access to quality healthcare services in remote areas. Their importance to the African continent stems from several factors: limited healthcare infrastructure, including a scarcity of well-equipped hospitals, trained professionals, and specialised treatment for complex diseases; frequent health crises, such as disease outbreaks (e.g., malaria, cholera, and Ebola) alongside chronic conditions and severe injuries; and significant geographic challenges, including natural disasters, conflicts, deserts, remote islands, and difficult terrain that obstruct healthcare accessibility [3,5].

Critical care transport (CCT) provides specialised medical care for patients with life-threatening conditions, facilitating their relocation from facilities with inadequate resources to fully equipped facilities [[5], [6], [7], [8], [9]]. Common medical conditions necessitating air ambulance transport in Africa include complex malaria, meningitis, pneumonia, intracranial haemorrhage, complications in premature infants, pregnancy complications, diving-related conditions, and trauma resulting from animal and road accidents [3,5,10].

Resilient critical care transport professionals (CCTPs) demonstrate proficiency in stress management, informed decision-making, and the delivery of high-quality care, thereby enhancing patient outcomes [[6], [7], [8], [9], [10]]. Resilience promotes cognitive flexibility and problem-solving capabilities, facilitating rapid and effective decision-making [9,[11], [12], [13], [14]]. Furthermore, resilience contributes to heightened job satisfaction and engagement, which, in turn, reduces turnover rates and aids in retaining experienced professionals [14,15]. Conversely, a deficiency in resilience amongst CCTPs can lead to adverse outcomes, such as burnout [4,[13], [14], [15]]. Implementing training, support, and well-being initiatives to foster resilience can mitigate these negative consequences and ensure high-quality patient care [[13], [14]]. Given the limited data concerning resilience within this specific environment, a research gap has been identified, warranting further exploration of this unique context. This study aimed to determine the resilience of CCTPs in a fixed-wing air ambulance environment in South Africa.

## Methods

The researcher engaged with the five largest fixed-wing air ambulance services in South Africa to maximise the number of participants meeting the inclusion criteria. These services operate from South Africa for both domestic and international air ambulance flights and represent >80 % of fixed-wing transfers. These transfers primarily originate from sub-Saharan Africa, the surrounding islands, India, and Europe. Transfer durations ranged from 10 to 36 h, depending on logistical factors, with patient contact times extending up to 18 h (and, in extreme circumstances, up to 24 h). The spectrum of diagnoses includes minor and major trauma, cardiac, neurology, medical and infectious diseases [[16], [17], [18], [19], [20]]. CCTP teams comprised either a flight doctor and flight nurse, a flight doctor and flight paramedic, or two flight paramedics [[16], [17], [18], [19], [20]].

Data collection occurred over a four-month period from June to September 2024. Owing to the geographical distribution of the study’s potential participants, they were introduced to the study during monthly flight meetings and via electronic communications. Informed consent was obtained prior to participation. Participants completed a survey incorporating the Connor-Davidson Resilience Scale to assess their resilience in the fixed-wing air ambulance environment [21]. Demographic information, including years of professional experience, team dynamics, and highest qualifications, was also collected. Participants completed the surveys at their convenience, enabling them to respond honestly and openly without external influence.

An exploratory descriptive research design was employed to evaluate the resilience of CCTPs in this environment [22]. To maintain participant confidentiality, no identifying information was included, and each participant was assigned a unique identification number. The completed survey guides were submitted to the study leader to maintain participant confidentiality [22].

This study utilised a survey incorporating the Connor-Davidson Resilience Scale to determine the resilience levels of CCTPs operating in a fixed-wing air ambulance environment [20]. An exploratory descriptive research design was employed to evaluate CCTPs’ resilience in this environment [22]. The survey introduced the study's purpose and objectives, provided a definition of resilience, and included a 25-item. Likert scale to assess CCTPs’ resilience level in a fixed-wing air ambulance environment [21]. Ethical clearance (Ref. 90/2024) was obtained from the University of Pretoria, Pretoria, South Africa.

Total population sampling techniques were employed [22]. This study involved recruiting CCTPs who met the inclusion criteria. The researcher purposefully selected CCTPs working in a fixed-wing air ambulance environment.

The dataset was captured in Microsoft Excel and analysed using IBM SPSS Statistics (version 30). Descriptive statistics were computed for the demographic variables, including frequency, mean, median, standard deviation, skewness, and kurtosis. The Shapiro–Wilk test was used to assess the normality of the data. The Shapiro–Wilk test assessed the normality of the data, revealing a violation of this assumption for the variables of interest (*p* < 0.05). Consequently, the nonparametric Kruskal–Wallis H test was employed to examine whether the distributions of scores differed significantly across the categories of demographic variables. Statistical significance was determined using an alpha level of 0.05. Pairwise comparisons were performed when significant differences were found, and p-values were adjusted for multiple tests using the Bonferroni correction. No missing values were found.

## Results

Responses were obtained from 48 CCTPs out of a possible 63, representing a response rate of 76 %. This depicts a response rate of 76 %. The majority of participants (*n* = 29, 60.4 %) were flight paramedics, followed by flight nurses (*n* = 12, 25.0 %) and flight doctors (*n* = 7, 14.6 %). The largest group of participants had less than five years of professional experience (*n* = 19, 39.6 %). An equal proportion of participants (*n* = 11, 22.9 %) had work experience ranging from five to ten years and ten to 20 years. The smallest group, (*n* = 7, 14.6 %), had more than twenty years of experience. Participants' qualifications included bachelor's degrees (*n* = 21, 43.8 %), diplomas/certificates (*n* = 16, 33.3 %), master's degrees (*n* = 10, 20.8 %), and other qualifications (*n* = 1, 2.1 %) (Table 1).

**Table 1:** Demographics.

Demographics	Categories	N	%
Profession	Doctor	7	14.6
	Nurse	12	25.0
	Paramedic	29	60.4
Total		**48**	**100**
Worked in this environment	< 5 years	19	39.6
	5–10 years	11	22.9
	> 10 years	7	14.6
	> 20 years	11	22.9
Total		**48**	**100**
Highest qualification	Bachelor’s degree	21	43.8
	Diploma/Certificate	16	33.3
	Master’s degree	10	20.8
	Other	1	2.1
Total		**48**	**100**

The findings indicate that participants generally exhibited resilience. Further details are provided in Table 2. Mean scores for the CD-RISC subscales ranged from 6.52 to 22.81, with standard deviations ranging from 1.07 to 3.44**.** Median values ranged from 6.0 to 23.0. For the total CD-RISC, the mean score was 79.04 (SD = 10.21), and the median was 80.0**.**

**Table 2:** Descriptive Statistics.

	Mean	Median	Standard Deviation	Minimum	Maximum	Skewness	Kurtosis
Hardiness	22.8125	23.0000	3.43732	13.00	28.00	−0.618	0.288
Coping	15.9167	16.0000	1.99823	9.00	19.00	−1.133	2.125
Adaptability/Flexibility	9.7917	10.0000	1.67533	3.00	12.00	−1.384	4.419
Meaningfulness/ Purpose	11.8750	12.0000	3.14626	3.00	16.00	−0.894	0.677
Optimism	5.7292	6.0000	1.75935	0.00	8.00	−0.986	1.317
Regulation of emotion and cognition	6.3958	6.0000	1.06670	4.00	8.00	−0.210	−0.443
Self-Efficacy	6.5208	7.0000	1.38364	2.00	8.00	−1.679	3.754
CD-RISC	79.0417	80.0000	10.20629	48.00	98.00	−0.514	0.752

*Abbreviations: CD-RISC: Connor-Davidson Resilience Scale.

**Fig. 2:**
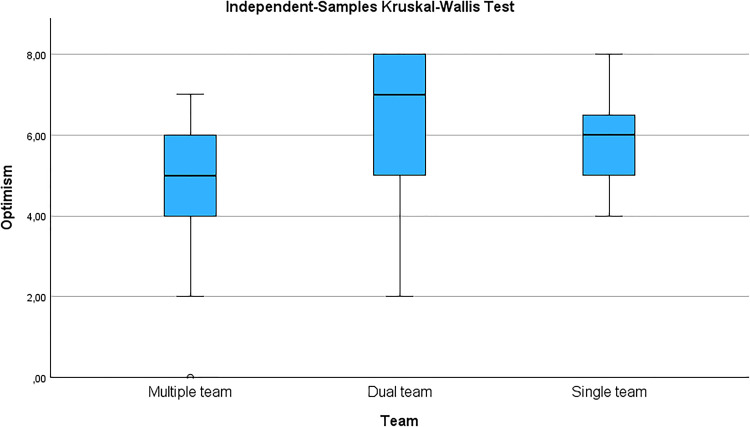
Optimism vs team composition (All values represent p-values from Kruskal-Wallis H tests).

Seven constructs exhibited no significant differences regarding team demographics, including composition, experience, qualifications, or profession (Table 3). A significant difference was identified in optimism and team composition; specifically, variations in optimism were observed between teams (*p* = 0.025). Teams composed of CCTPs from multiple professions demonstrated higher mean scores, followed by teams composed of CCTPs from the same profession (Fig. 2).

**Table 3:** Constructs of resilience.

	Profession	Experience	Highest qualification	Team
Hardiness	0.721	0.064	0.926	0.636
Coping	0.413	0.793	0.989	0.708
Adaptability/Flexibility	0.278	0.269	0.399	0.885
Meaningfulness Purpose	0.508	0.117	0.906	0.597
Optimism	0.227	0.559	0.158	0.025
Regulation of Emotion and cognition	0.423	0.382	0.316	0.573
Self-Efficacy	0.788	0.600	0.943	0.616
CD-RISC	0.486	0.213	0.977	0.463

*Abbreviations: CD-RISC: Connor-Davidson Resilience Scale.

The distribution of hardiness, coping, adaptability, meaningfulness and purpose, emotion regulation, and self-efficacy remained similar across all the demographic variables.

Post-hoc pairwise comparisons, conducted using Dunn–Bonferroni tests, revealed a statistically significant difference between the multiple and dual team groups (*p* = 0.020). However, no significant differences were observed between the multiple and single-team groups (*p* = 0.765) or between the single and dual-team groups (*p* = 0.671).

## Discussion

This study found that CCTPs working in South Africa's fixed-wing air ambulance environment demonstrated high overall resilience, with no significant differences observed across professional categories or years of experience. However, optimism was significantly higher amongst mixed-profession teams, suggesting that team composition may influence certain resilience dimensions. Each of the seven constructs is discussed as an important contributor to resilience amongst CCTPs.

**Hardiness**, a personality trait characterised by commitment, control, and challenge, plays a key role in CCTP resilience and stress management. Velando-Soriano et al. (2020) indicate that psychological hardiness can significantly impact occupational stress and burnout amongst healthcare workers in critical care settings. A study of critical care nurses demonstrated that hardiness predicted occupational stress and burnout, with "commitment to work" being particularly influential in mitigating burnout [[23], [24]]. In this study, CCTP participants had a mean hardiness score of 22.8, indicating high hardiness. Hardiness mediates the effects of emergency stress on emotional exhaustion and depersonalisation aspects of burnout. For healthcare workers during high-pressure situations such as CCT, hardiness and positive coping strategies are crucial for reducing stress-related burnout [25]. Furthermore, enhancing hardiness involves developing strategies that bolster commitment, control, and the perception of challenges [26]. For CCTP participants, these characteristics are fundamental for maintaining psychological health and performance under pressure.

**Coping** refers to the strategies and behaviours individuals employ to manage stress, address challenges, or alleviate emotional distress [27,28]. Coping strategies support cognitive-constructive therapy protocols in managing stress and reducing burnout, compassion fatigue, and health issues [28]. CCTPs, similar to emergency medical service professionals, frequently experience high emotional exhaustion and diminished personal achievement [27]. Strategies such as discussing concerns with colleagues, anticipating periods of rest, and reflecting on positive impacts can vary emotional exhaustion and depersonalisation [27]. In this study, CCTPs demonstrated a mean coping score of 15.9, indicating problem-solving and positive coping within CCT, which predicted higher compassion satisfaction and mitigated secondary stress syndromes [28]. Interventions promoting positive coping enhance care quality. Supportive work environments and social support alleviate stress and improve job satisfaction [28,29]. Integrating diverse coping strategies, including problem-focused, emotion-focused, and supportive mechanisms, maintains CCTPs well-being, aids in stress management, and contributes to occupational health and service quality [30].

**Adaptability and flexibility** are essential CCT traits due to dynamic medical emergencies [31]. These professionals operate in high-pressure environments where rapid adaptation impacts patient outcomes [31]. Adaptability refers to adjusting responses and strategies to meet patient needs, including treatment protocols and technology [7]. A mean score of 9.79 indicates that flexibility involves modifying approaches within established frameworks to optimise performance, such as rerouting transport due to weather or traffic [31]. Given Africa's limited resources and poor infrastructure, the use of CCTPs is necessary. Human resource flexibility is crucial for fostering adaptability and influencing innovation and challenge management, thereby enabling healthcare systems to effectively manage patient transportation [31,32]. In Africa, CCTPs' adaptability is vital due to healthcare and resource challenges. This study demonstrates that CCTPs can adapt to resource limitations and provide care across vast areas without sophisticated infrastructure.

**Meaningfulness** is the quality or state of having significance, purpose, or value [33]. The presence of meaningfulness in CCTPs enhances resilience by influencing the personal well-being, motivation, and patient outcomes of healthcare professionals in emergency environments [33]. The resilience of CCT systems can be improved through strategies that incorporate system-level and individual interventions [33]. CCTPs scored a median of 1.87 for meaningfulness, improving their psychological well-being and resilience, helping them manage distress, and improving patient care [33,34].

**Optimism** is a mental attitude characterised by the expectation that good things will happen [35],[35]. For healthcare workers, optimism reduces stress and improves outcomes by buffering stressors and boosting resilience [35]. Optimistic healthcare workers demonstrate enhanced problem-solving skills and proactively seek support, thereby improving their task and crisis management abilities [35]. Furthermore, an optimistic outlook fosters supportive relationships when transporting critically ill or injured patients [36]. In this study, the CCTPs scored a median of 5.72 for optimism. Optimism was the only construct that showed a significant difference between mixed and same-profession team compositions. This indicates that optimism can be enhanced in teams with multiple professions. Optimism and resilience mitigate anxiety and depression in high-pressure environments [36]. Developing optimism enhances resilience within critical care settings by utilising strength-based approaches and optimism-building programs for CCTPs, which are valuable for both daily operations and emergency preparedness [36]. In summary, fostering optimism is essential for resilient CCTPs to thrive. By fostering a positive outlook, CCTPs can enhance their effectiveness under challenging conditions.

**Regulation of emotion** is crucial for enhancing resilience amongst CCT professionals, as suggested by Polizzi (2023). This demonstrates the benefits of fostering psychological resilience under stress. It is linked to managing adversity, facilitating problem-focused coping, and maintaining healthy functioning despite challenges [37]. In critical care transport, effective emotion regulation sustains resilience and mental well-being [37,38]. Higher resilience correlates with improved mental health, enabling effective stress management and mitigating the risk of depression or burnout [38]. This resilience is mediated by emotion regulation. This resilience is mediated by emotion regulation; emotional regulation training benefits intensive care professionals by improving their psychological well-being and reducing burnout [39]. Specifically, for CCTPs, daily challenges highlight the need for resilient coping mechanisms amongst African CCTs. Cognitive emotion regulation buffers stress, as individuals learn to positively reappraise situations. The ability to regulate emotions mediates resilience and distress. Intervention strategies targeting emotion regulation have increased resilience in healthcare environments [40], highlighting their potential in critical care.

**Self-efficacy** refers to an individual's belief in their capacity to perform a task or achieve a goal [41]. CCTPs face high-pressure situations that require quick decisions and emotional endurance. Research indicates a link between resilience and professional self-efficacy in healthcare, mediated by ethical vision and support [41]. These factors are particularly salient in critical care and present significant ethical challenges. CCTPs experience moral distress, which can be mitigated through the development of moral resilience [42]. This resilience, characterised by integrity, buoyancy, and self-regulation, aids in addressing ethical dilemmas [[42], [43]]. CCTPs demonstrated a median self-efficacy score of 6.52. Their resilience is influenced by growth opportunities, organizational support, and teamwork. Management strategies focused on psychological capital and support cultivate resilience and enhance job involvement and care quality [44]. Studies during the COVID-19 pandemic have highlighted the association between resilience and self-efficacy among healthcare workers [44]. Resilience programs bolster both constructs, facilitating stress management and improving quality of life [[41], [45]].

## Limitations

The perspectives of the participants may not be representative of those of other CCTPs in South Africa or internationally, thereby limiting the transferability of these findings. Due to the voluntary nature of participation, a self-selection bias was likely present. Nevertheless, the participants were broadly distributed across South Africa and were situated in diverse fixed-wing environments, resulting in a range of perspectives. Furthermore, the transferability of these perspectives is discussed in relation to similar studies conducted in different contexts. The proportion of air ambulance services based in Johannesburg (*n* = 4) was greater than that of services located elsewhere (*n* = 1), introducing a sampling bias. Additionally, this study assumed that individuals completing the survey accurately reported the characteristics of their experiences, which may have introduced reporting bias into the results.

## Conclusion

This study aimed to determine the level of resilience amongst CCTPs in the fixed-wing air ambulance environment in SA. The importance of the seven constructs–Hardiness, Coping and Adaptability, Flexibility, Meaningfulness and Purpose, Optimism, Regulation of Emotions, and Cognition and Self-Efficacy–was assessed, revealing a high level of resilience among CCTPs. Resilience is a vital characteristic for CCTPs, ensuring effective teamwork, communication, and quality care, ultimately leading to improved patient outcomes. Optimism is depicted as one of the most important attributes of professional team composition. It is strongly suggested of a multi-professional team composition; therefore, suggesting that the team composition of a multi professional composition should be encouraged. Suggestions, training, and support to enhance these 7 constructs and support CCTPs should be investigated, and pilot studies exploring these suggestions should be conducted.

The findings of this review provide a basis for supporting CCTPs resilience, recognising the challenges and needs of CCTPs in this challenging environment is foundational for future research, policy, and practice.

Future research should investigate resilience amongst CCTPs across diverse air ambulance environments, extending beyond fixed-wing operations. Additionally, this study did not specifically address gender differences; therefore, subsequent research should explore the distinctions and commonalities between male and female CCTPs.

## Dissemination of results

The results of this study were shared with CCTPs and staff members at the data collection sites via an informal presentation at either their monthly flight meetings or through electronic communication groups.

## Declaration of generative AI and AI-assisted technologies in the manuscript preparation process

The author utilised Paperpal for language and editing assistance during the preparation of this work. After using this tool/service, the author(s) reviewed and edited the content as needed and take(s) full responsibility for the content of the published article.

## Funding information

No funding was received by authors for the publication

## CRediT authorship contribution statement

**Karien Basson:** Data curation, Methodology, Investigation, Conceptualization, Writing – review & editing, Writing – original draft. **Isabel Coetzee-Prinsloo:** Data curation, Conceptualization, Methodology, Writing – review & editing, Writing – original draft, Supervision. **Annatjie van der Wath:** Conceptualization, Methodology, Writing – review & editing, Writing – original draft, Supervision. **Andries Masenge:** Data curation, Conceptualization, Writing – review & editing, Writing – original draft.

## Declaration of competing interest

The authors declare that they have no known competing financial interests or personal relationships that could have appeared to influence the work reported in this paper.
